# Comparison of Intraoperative Parameters between Two Handpieces in One-Handed Phacoemulsification with Active Fluidics

**DOI:** 10.3390/diagnostics14192141

**Published:** 2024-09-26

**Authors:** Jerónimo Araño-Ferrer, Ana Beatriz Medina-Perez, Cyntia Solis-Hernandez, Rosario Gulias-Cañizo, Oscar Guerrero-Berger

**Affiliations:** 1 Department of Anterior Segment Surgery, Fundación Hospital Nuestra Señora de la Luz, Mexico City 06030, Mexico; jeronimoaranof@hotmail.com (J.A.-F.); cynsolishernandez@gmail.com (C.S.-H.); 2 Centro Oftalmológico Mira, Mexico City 06760, Mexico

**Keywords:** anterior chamber stability, Active Sentry, Ozil, phacoemulsification, phaco handpiece, surge, IOP sensor

## Abstract

**Purpose**: To compare anterior chamber stability and surgical efficiency in one-handed phacoemulsification, comparing Ozil and Active Sentry (AS) handpieces. **Methods**: Observational and comparative study. Selected patients were divided into two groups, AS and Ozil handpieces, and underwent one-handed phacoemulsification. Parameters like IOP per quadrant, vacuum, and aspiration flow were fixed in all surgeries. The study endpoints were intraoperative anterior chamber instability score (IACIS), cumulative dissipated energy (CDE), followability, and surgery duration. **Results**: Mean age was 71.42 years in the AS group vs. 73.97 in the Ozil Group. Mean Axial Length was 22.85 ± 1.21 mm with Active Sentry vs. 23.3 ± 1.29 mm with Ozil (*p* = 0.324). IACIS was 0.10 ± 0.30 with AS vs. 0.63 ± 0.71 with Ozil (˂0.001*). CDE was 9.95 ± 4.76 percent-seconds with AS vs. 10.89 ± 6.55 percent-seconds with Ozil (0.519). The followability score was 0.74 ± 0.855 with AS vs. 0.83 ± 0.874 with Ozil (*p* = 0.678). Surgery duration was 19.00 ± 5.44 min with AS vs. 24.57 ± 6.51 with Ozil (*p* < 0.001). **Conclusions**: The Active Sentry handpiece improves anterior chamber stability in one-handed phacoemulsification while maintaining surgical performance during nucleus removal without an auxiliary side-port. To the best of our knowledge, this is the first study demonstrating that the Active Sentry handpiece can increase anterior chamber stability not only in conventional phacoemulsification but also in one-handed phacoemulsification.

## 1. Introduction

One-handed phacoemulsification is a technique that has gained momentum as it is a less invasive surgery compared to bimanual surgery. The reduced invasiveness implies less biomechanical modification and, consequently, less induction of post-operative astigmatism, which is very relevant since one goal of phacoemulsification is refractive success. For example, some studies indicate that a second incision has a rotating effect on the axis of astigmatism [1], and even though both one- and two-handed techniques can decrease total corneal astigmatism, the angle of error, that is, the difference between surgically induced astigmatism and target-induced astigmatism, is significantly smaller after one-handed phacoemulsification [2]. Other reported advantages of one-handed phacoemulsification are that it induces less endothelial cell loss [3,4] and has better early visual outcomes compared to the bimanual technique [3]. Given all the benefits of one-handed phacoemulsification, it is relevant to understand which systems allow an adequate performance of this technique.

**Figure 1:**
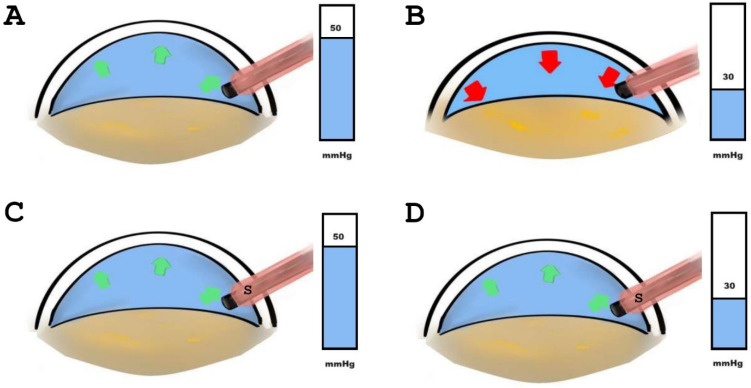
Fluidic compensation after lowering irrigation pressure from 50 to 30 mmHg (**A**,**B**) does not avoid anterior chamber (AC) instability compared to the handpiece that includes a sensor (**C**,**D**) that adjusts pressure fluctuations more quickly and efficiently, consequently reducing the frequency and severity of surge. (Green arrows represent AC stability; red arrows represent AC instability.

Hydrodynamic pressure is an essential parameter during cataract surgery since one of the intraoperative challenges of phacoemulsification is the risk of surge. Currently, there are two fluidic systems for phacoemulsification machines equipped with peristaltic pumps: gravity-fluidics and active-fluidics (Alcon Laboratories, Fort Worth, TX, USA). Infusion pressure in gravity-fluidics is mainly determined by the bottle height, adjusted based on the surgeon’s feedback. On the other hand, infusion pressure in the active-fluidics system depends on pressure sensors that monitor intraocular pressure changes. These sensors are located either in the cassette (for the Ozil handpiece) or the handpiece (for the Active Sentry handpiece), the latter representing an additional anti-surge technology that potentially maintains consistent intraoperative parameters. Anti-surge technology is relevant for the efficiency and safety of phacoemulsification, since surge, the abrupt reduction of positive pressure within a fluidic system, may cause intraoperative complications. Specifically, the Centurion^®^ Vision System (Alcon Laboratories, Fort Worth, TX, USA) may include the Active Sentry handpiece with an integrated pressure sensor, unlike other handpieces such as the Ozil handpiece. The ability of Active Sentry to sense IOP at the level of the handpiece allows a faster compensation of cumulative vacuum after occlusion break, decreasing the risk of surge [5]. In an experimental model, the use of the Active Sentry system demonstrated less aqueous volume losses after occlusion break versus other systems [6], and less surge duration and volume while maintaining anterior chamber depth even during occlusion breaks [7]. Both phacoemulsification energy and time are among the most critical factors affecting corneal endothelial cell loss, where the Active Sentry system has shown less cumulative dissipated energy (CDE) delivery, with shorter ultrasound duration [8]. Another recently reported advantage derived from the lower infusion pressure with the Active Sentry system is that it has fewer effects on the hyaloid membrane barrier than the Ozil system, which induces changes in the vitreous-lens interface [9]. Figure 1 shows the risk of surge related to pressure depending on the presence of a sensor in the handpiece.

**Figure 2:**
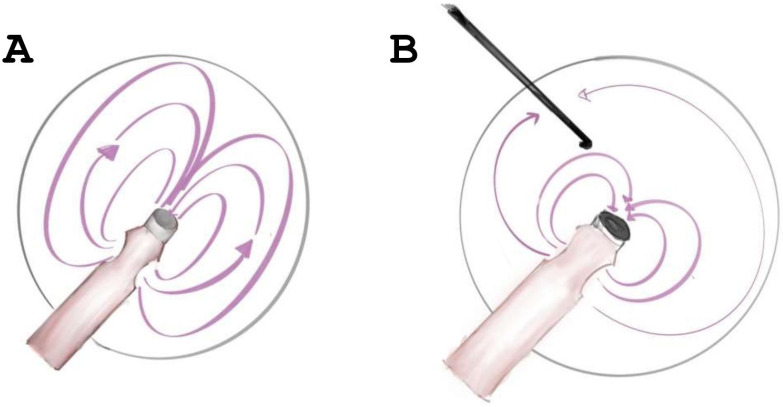
Figure legend: one-handed phacoemulsification (**A**) delivers a greater hermeticity of the anterior chamber, observing that currents have fewer fluidic vices since there is no side port leakage. In contrast, the presence of a side port (**B**) draws currents toward it, leading to additional incisional leakage.

In bimanual surgery, regardless of the system and handpiece used, there is fluid leakage either directly at the main incision through the handpiece sleeve or through the accessory port that may have unaccounted outputs that alter the fluidic balance. Hydrodynamic pressure varies when any system is evaluated with bimanual surgery, making it more challenging to validate its performance. However, removing the variable of a second port makes it easier to estimate the true hydrodynamic effect of the pressure sensor in the handpiece. Since one-handed phacoemulsification has better control of hydrodynamic status because it resembles a closed chamber with minimum fluid leakage (Figure 2) [10], we aimed to compare anterior chamber stability and surgical efficiency with this technique using the Ozil and Active Sentry handpieces.

## 2. Material and Methods

This observational, single-center, and comparative study adhered to the latest tenets of the Declaration of Helsinki. It was approved by the Ethics in Research Committee of the Instituto de Servicios Periciales y Ciencias Forenses (approval number 029/2023). We screened consecutively all patients older than 50 years eligible for routine phacoemulsification with a diagnosis of cataract LOCS III grades 2–3 until we reached the estimated sample size for the study. Exclusion criteria included age < 50 years and >75 years, diagnosis or history of intraocular surgery, any inflammatory ocular disorder, glaucoma, pseudoexfoliation, pupil dilation < 6 mm, ocular trauma, zonular dysfunction, including zonular laxity, lens subluxation or dislocation, or traumatic cataract. All patients provided informed consent for study participation and were randomized into one of two groups: Active Sentry or Ozil. 

One experienced surgeon performed all the surgeries using a one-handed technique, as follows. After a 2.2-mm clear corneal incision followed by ophthalmic viscoelastic injection (DisCoVisc^®^, hyaluronic acid 1.6%-chondroitin sulfate 4.0%, Alcon Laboratories), capsulorhexis, hydrodissection, and nuclear rotation, phacoemulsification using the Centurion^®^ Vision System (Alcon Laboratories, Fort Worth, TX, USA) was initiated using the divide and conquer technique. After the nucleus was segmented into four quadrants using a prechopper, phacoemulsification was continued with fixed parameters: IOP (1st quadrant 50 mmHg, 2nd quadrant 46 mmHg, 3rd quadrant 40 mmHg, and 4th quadrant 36 mmHg), aspiration flow rate (40 cc/min), vacuum (500 mmHg), and 70% torsional ultrasound with continuous linear mode. After nucleus removal, the surgeon performed cortex aspiration, IOL implantation, thorough viscoelastic aspiration, and sutured the phaco incision with 10–0 nylon. During surgery, the position of the iris-lens diaphragm was observed at all times both by the surgeon and the study co-ordinator, who captured all the data to obtain the primary endpoint of the study, the intraoperative anterior chamber instability score (IACIS) (modified from Geng W et al.) [11]. This score was obtained by observing the position of the iris-lens diaphragm under the operating microscope to determine anterior chamber changes, counting for each procedure the number of times that each event occurred, with the following scale:

- Zero points: absence of forward movement of the posterior capsule during nuclear removal, lack of forward movement of the iris–lens diaphragm during nuclear removal, and no pupil diameter fluctuations.
- One point: Forward movement of the posterior capsule during nuclear removal, forward movement of the iris-lens diaphragm during nuclear removal, or pupil diameter fluctuations.
- Two points: Capsule aspiration without posterior capsule rupture during nuclear removal.

Also, CDE, measured in percent-seconds, was captured as a secondary endpoint, and both followability (graded based on the number of nuclear repulsion events that required tip-reacquisition movements from the surgeon) and surgery duration were included as exploratory endpoints.

### Statistical Analysis

Data were captured in an Excel spreadsheet (Microsoft Office, Microsoft Corporation, Redmond, WA, USA) without identifying patient data and transferred to an SPSS (v30.0.0) file (SPSS Statistics, IBM, Armonk, NY, USA) for statistical analysis. Numerical variables were described with central tendency and dispersion measures (standard deviation (SD)) and categorical variables with absolute numbers and percentages. The correlation between categorical variables was evaluated using the Chi-square test. Numerical variables were analyzed with the Kolmogorov–Smirnov test to determine whether their distribution was parametric or non-parametric. In the case of parametric distribution, the relationship between numerical and categorical variables was analyzed using the Student’s *t*-test. For non-parametric distributions, the difference in the distribution of numerical variables in independent groups was evaluated with the U-Mann–Whitney test. 

## 3. Results

We enrolled 61 eyes in both groups, 31 in the Active Sentry group and 30 in the Ozil group. Table 1 describes demographic variables. The proportion of right and left eyes differed between groups, with the Active Sentry having more left eyes and the Ozil having more right eyes. However, this difference did not reach statistical significance (*p* = 0.054). The mean age was similar for the Active Sentry and Ozil groups (71.42 and 73.97 years, respectively; *p* = 0.324). Gender distribution was also comparable between groups (*p* = 0.893). Axial length showed no statistically significant differences (*p* = 0.544). The tables do not include all fixed parameters (mentioned in methods) like aspiration flow (40 cc/min), IOP per quadrant, and vacuum.

Table 2 shows endpoint comparisons between groups. There were no statistically significant differences between mean CDE in the Active Sentry and Ozil groups (9.94 vs. 10.89 percent-seconds, *p* = 0.519, respectively). On the other hand, the mean surgery duration was lower with Active Sentry than with Ozil (19.00 vs. 24.57 min, *p* = 0.001). When comparing the followability score, there were no statistically significant differences between groups (*p* = 0.678). On the other hand, when comparing the mean IACI score, patients in the Active Sentry group had lower values (0.10) than Ozil patients (0.63; *p* ≤ 0.001). This significance persisted when treating the IACI score as a categorical variable, where the Active Sentry group only had IACI instances of 0 and 1. In contrast, the Ozil group had cases of 0, 1, and 2 (*p* = 0.002). 

## 4. Discussion

Over the years, developing different anti-surge mechanisms has helped to reduce complications in phacoemulsification. These mechanisms are surgeon-dependent or independent. Among the dependent ones are the type and amount of ophthalmic viscoelastic used, and the configuration of irrigation pressure, vacuum, and aspiration flow. Regarding independent variables, the most relevant technological advances were the modification of the materials of the phaco tubes to reduce compliance, the inclusion of venting systems to decrease the negative pressure of the hydrodynamic system, and the development of increasingly precise intraocular pressure sensors located in different locations of phacoemulsification machines, until today where we have a system considered the most advanced one with a vacuum sensor located in the handpiece (Active Sentry) of the Centurion Vision System.

This study evaluated both anterior chamber stability and surgical efficiency. Anterior chamber stability reflects the compensatory mechanisms’ efficiency when hydrodynamic variations occur. Our results showed that the IACI score, used to measure anterior chamber stability in an objective way [11], was significantly lower when using the Active Sentry handpiece in a context of low hydrodynamic variation due to minimal fluid losses, namely one-handed phacoemulsification. Additionally, we evaluated surgical efficiency through surgery duration, CDE, and followability.

Surgery duration is crucial since prolonged surgical times increase the risk of endothelial cell loss, inflammation, and endophthalmitis [12,13,14]. Active Sentry reduced surgical duration statistically significantly, mainly because it reduces pauses due to intraoperative pressure fluctuations. Regarding CDE and followability, we did not observe any differences between groups. CDE is the amount of ultrasound delivered in a time frame and is generally proportional to the hardness of the nucleus. In this study, we included LOCS III grade 2–3 cataracts in both groups, so finding no differences in this parameter is unsurprising. However, some studies have reported less CDE delivery with Active Sentry [8]. We believe that we did not observe differences in this parameter because we set the vacuum at 500 mmHg, a moderate vacuum value that, if higher, would have decreased CDE. Similarly, aspiration flow was set at 40 cc/min. Had aspiration flow been set higher, CDE would have been lower.

Since there is no side-port incision in one-handed phaco, followability is essential to maintain surgical efficiency, reducing undesirable fluidic circuits. Our results did not show significant differences in the followability score between both handpieces because followability depends more on the surgical technique than on the handpiece used. The intention of including followability as a variable for evaluation in this study was to verify the preservation of followability compared to conventional bimanual phacoemulsification since, to the best of our knowledge, this is the first study that evaluates one-handed phacoemulsification with the Active Sentry handpiece.

One of the limitations of our study is that neither the surgeon nor the observer were blind to the study groups, since both handpieces are different, allowing for easy identification. However, the observer was not aware of the study design, decreasing bias.

Finally, our results show that surgery duration was shorter in the Active Sentry group. We believe this is due to enhanced anterior chamber stability and preservation of followability since both parameters improve hydrodynamic efficiency, making it easier for an experienced surgeon to perform a fluid surgery with minimal or no adjustment of intraoperative parameters.

## 5. Conclusions

- The Active Sentry handpiece improves anterior chamber stability in one-handed phacoemulsification, as the IACI score demonstrates.
- There were no differences in followability between groups, indicating that the Active Sentry handpiece maintains surgical performance during nucleus removal without an auxiliary side-port.
- To the best of our knowledge, this is the first study demonstrating that the Active Sentry handpiece can increase anterior chamber stability in conventional and one-handed phacoemulsification.

## Figures and Tables

**Table 1:** Demographic variables.

Variable	Active Sentry	Ozil	*p*-Value
Age (Years) ^¥^	71.42 ± 10.535	73.97 ± 9.434	0.324
Gender (Male/Female) ^ǂ^	51.6/48.4	53.3/46.7	0.893
Laterality (Right/Left Eye) ^ǂ^	38.7/61.3	63.3/36.7	0.054
Axial Length (mm) ^¥^	22.84 ± 1.21	23.30 ± 1.29	0.544

^ǂ^ Results expressed as percentages; ^¥^ results expressed as means ± SD.

**Table 2:** Outcomes per group.

Endpoint	Active Sentry	Ozil	*p*-Value
Surgery duration (minutes) ^ǂ^	19.00 ± 5.447	24.57 ± 6.516	0.001 *
Followability score ^ǂ^	0.74 ± 0.855	0.83 ± 0.874	0.678
Intraoperative anterior chamber instability score ^ǂ^	0.10 ± 0.30	0.63 ± 0.71	˂0.001 *
Cumulative dissipated energy (percent-seconds) ^ǂ^	9.95 ± 4.76	10.89 ± 6.55	0.519

^ǂ^ Results expressed as means ± SD; * statistically significant.

## Data Availability

The original contributions presented in the study are included in the article, further inquiries can be directed to the corresponding author/s.
